# The maximal metabolic steady state: redefining the ‘gold standard’

**DOI:** 10.14814/phy2.14098

**Published:** 2019-05-23

**Authors:** Andrew M. Jones, Mark Burnley, Matthew I. Black, David C. Poole, Anni Vanhatalo

**Affiliations:** ^1^ Sport and Health Sciences University of Exeter St. Luke's Campus Exeter United Kingdom; ^2^ School of Sport and Exercise Sciences University of Kent Medway United Kingdom; ^3^ Department of Kinesiology Kansas State University Manhattan Kansas

**Keywords:** Fatigue, metabolism, performance

## Abstract

The maximal lactate steady state (MLSS) and the critical power (CP) are two widely used indices of the highest oxidative metabolic rate that can be sustained during continuous exercise and are often considered to be synonymous. However, while perhaps having similarities in principle, methodological differences in the assessment of these parameters typically result in MLSS occurring at a somewhat lower power output or running speed and exercise *at* CP being sustainable for no more than approximately 20–30 min. This has led to the view that CP overestimates the ‘actual’ maximal metabolic steady state and that MLSS should be considered the ‘gold standard’ metric for the evaluation of endurance exercise capacity. In this article we will present evidence consistent with the contrary conclusion: i.e., that (1) as presently defined, MLSS naturally underestimates the actual maximal metabolic steady state; and (2) CP alone represents the boundary between discrete exercise intensity domains within which the dynamic cardiorespiratory and muscle metabolic responses to exercise differ profoundly. While both MLSS and CP may have relevance for athletic training and performance, we urge that the distinction between the two concepts/metrics be better appreciated and that comparisons between MLSS and CP, undertaken in the mistaken belief that they are theoretically synonymous, is discontinued. CP represents the genuine boundary separating exercise in which physiological homeostasis can be maintained from exercise in which it cannot, and should be considered the gold standard when the goal is to determine the maximal metabolic steady state.

## Introduction

Knowledge of the running speed or cycling power output which generates the maximal sustainable oxidative metabolic rate may be important in appraising athletic performance potential and in guiding athletic training programs (Jones and Carter 2000; Morton 2006; Jones et al. 2010; Vanhatalo et al. 2011a). Performing training below, compared to above, such a threshold will invoke acute differences in oxidative and nonoxidative energy supply, muscle and blood biochemistry, cardiorespiratory responses, fatigue processes, and effort perception, which, if repeated chronically, would be expected to promote different physiological adaptations (Holloszy and Coyle 1984; Jones and Carter 2000). While this notion is widely accepted, a plethora of terms and techniques have emerged which purport to describe or determine this ‘maximal metabolic steady state’. For example, phenomena derived from incremental exercise tests which have been proposed to correspond to, or enable an accurate estimation of, maximal metabolic steady state include the lactate threshold (LT), gas exchange threshold (GET), ventilatory threshold, lactate turn‐point (LTP), anaerobic threshold, the ‘onset of blood lactate accumulation’ corresponding to an absolute blood lactate concentration ([lactate]) of 4 mmol/L (OBLA), individual anaerobic threshold, lactate minimum, and respiratory compensation threshold (Faude et al. 2009; Jones et al. 2018). Not only do these terms reflect very different physiological events and mechanisms, they may occur at contrasting metabolic rates and, to a degree, may be a function of the measurement technique or specific testing paradigm employed (Jamnick et al. 2018). Accordingly this has led to considerable confusion and misunderstanding in this field (see Jones et al. 2018, for detailed critique).

**Figure 1:**
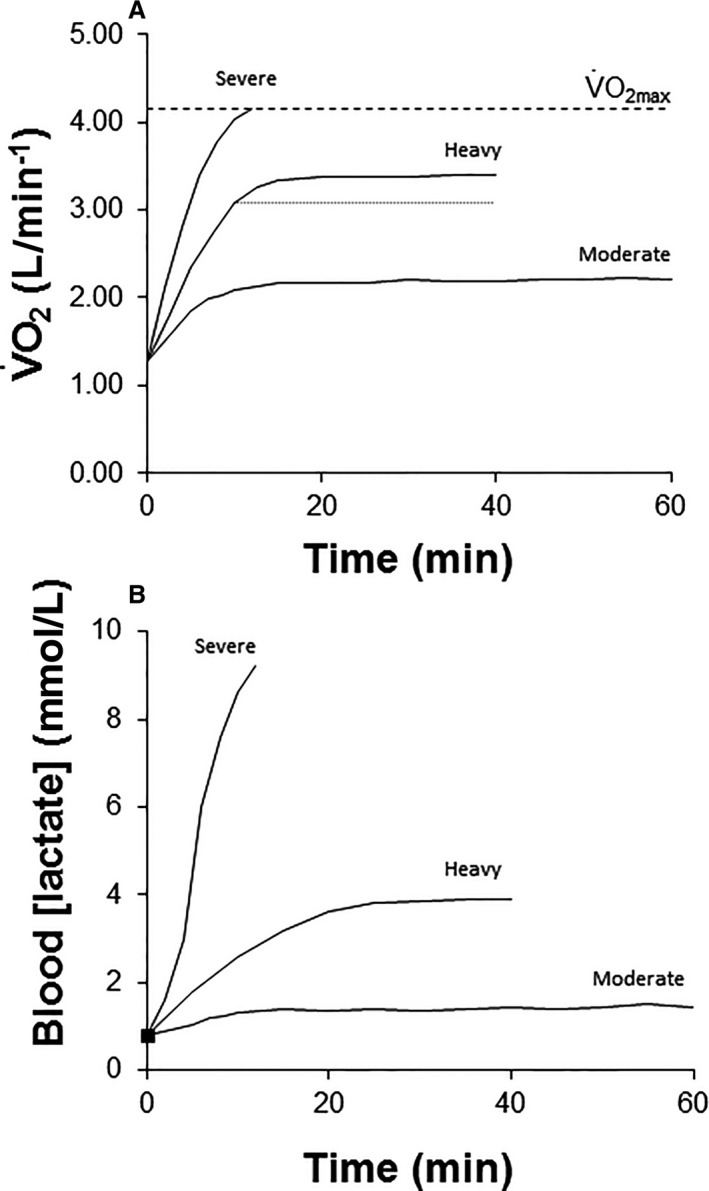
Schematic representation of the pulmonary oxygen uptake ($\dot{V}$O_2_) (panel A) and blood [lactate] responses (panel B) during moderate‐intensity, heavy‐intensity, and severe‐intensity exercise. During moderate‐intensity exercise, $\dot{V}$O_2_ and blood [lactate] reach steady‐state values rapidly. During heavy‐ and severe‐intensity exercise, there is an additional oxygen cost (termed $\dot{V}$O_2_ slow component) above that expected from the extrapolation of the moderate‐intensity $\dot{V}$O_2_‐power output relationship. During heavy‐intensity exercise, the attainment of (higher amplitude) steady‐state values for $\dot{V}$O_2_ and blood [lactate] is delayed. The magnitude of the $\dot{V}$O_2_ slow component during heavy‐intensity exercise is illustrated by the dotted line provided in panel A. During severe‐intensity exercise, $\dot{V}$O_2_ and blood [lactate] continue to rise until $\dot{V}$O_2_max (panel A, dashed line) is attained with the limit of tolerance occurring shortly thereafter.

As a first step towards greater clarity, it is essential to appreciate the existence of discrete exercise intensity domains within which the physiological responses to exercise differ considerably (Whipp and Ward 1992; Poole and Richardson 1997; Carter et al. 2002; Wilkerson et al. 2004; Black et al. 2017). The pulmonary O_2_ uptake ($\dot{V}$O_2_) and blood [lactate] responses to constant‐power moderate, heavy, and severe‐intensity exercise are schematized in Figure 1. These profiles indicate that the achievement of steady‐state values in these variables is markedly different in these discrete exercise domains (i.e., rapid in the moderate‐intensity domain, delayed in the heavy‐intensity domain, and not possible in the severe‐intensity domain). These differences reflect variable energy system contribution and have clear implications for fatigue development and exercise tolerance (Whipp and Ward 1992; Jones et al. 2011). Some identifiable ‘thresholds’ during incremental exercise demarcate the transition from moderate to heavy‐intensity exercise (i.e., LT and GET) whereas others purport to demarcate the transition from heavy to severe‐intensity exercise (i.e., LTP and, arguably, OBLA). The first threshold is relevant for ultra‐endurance and low‐intensity endurance events and in occupational and clinical physiology. However, it is the definition, evaluation, and application of the second boundary, which typically occurs at 75–90% $\dot{V}$O_2_ max and is therefore more relevant to most types of high‐level endurance exercise performance (Jones and Poole 2008; Jones and Vanhatalo 2017), that is the focus of this review.

**Figure 2:**
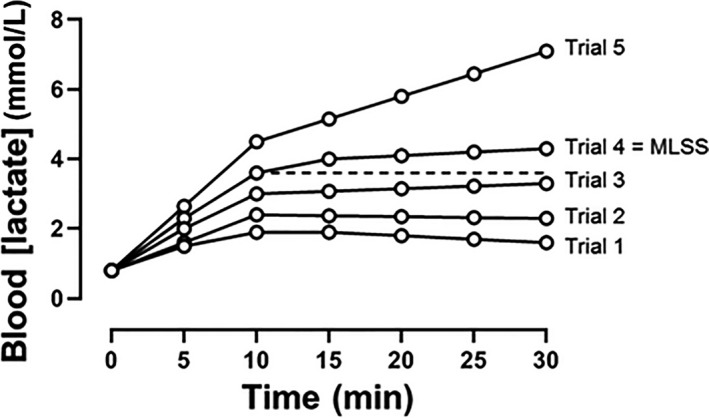
Schematic representation of the blood [lactate] response to a series of constant running speed tests performed on separate days for the determination of MLSS. Trial 1 is representative of the lowest running speed chosen and each trial is indicative of an increment in speed until trial 5 (the highest running speed applied). During trials 1, 2, 3, and 4, blood [lactate] does not increase by more than 1 mmol/L between minutes 10 and 30. However, during trial 5, blood [lactate] is 4.5 mmol/L at 10 min and 7.1 mmol/L at 30 min (Δ2.6 mmol/L). Therefore, in spite of a gradual increase (Δ0.7 mM) in blood [lactate] between minutes 10 and 30, trial 4 represents the highest running speed at which blood [lactate] did not rise by more than 1 mM ‐ and it would therefore be defined as MLSS. Note therefore that the *actual* MLSS, according to the accepted definition, lies at a speed somewhere between trial 4 and trial 5, such that the MLSS selected (trial 4) will necessarily be an underestimate. The dashed line is indicative of the blood [lactate] attained at 10 min during trial 4, and is projected to the end of the exercise trial.

**Figure 3:**
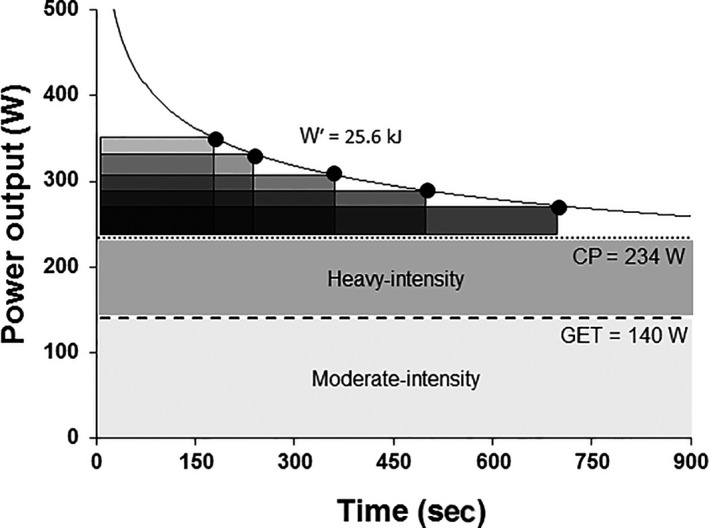
Schematic representation of the power‐duration relationship with reference to the moderate‐intensity (light gray shaded area), and heavy‐intensity (dark gray shaded area) exercise intensity domains. The boundary between the moderate‐ and heavy‐intensity domains is given by the lactate or gas exchange threshold (GET), and the boundary between the heavy‐ and severe‐intensity domains is given by the critical power (CP). The CP and the work capacity available above CP (termed Wʹ) can be determined using a series of constant power output trials performed to the limit of tolerance within the severe‐intensity domain (i.e., >CP). The CP is defined as the power asymptote (234 W in this example), and Wʹ is characterized by the curvature constant (25.6 kJ in this example), of this hyperbolic relationship between power output and time. The Wʹ is capacity‐, but not rate‐, limited and therefore its contribution (in kJ) to severe‐intensity exercise is constant irrespective of exercise duration in the severe‐intensity domain. The greater the difference between the power output being sustained and CP, the more rapidly W′ will be utilized, with the limit of tolerance coinciding with the exhaustion of W′. The hyperbolic relationship between power and time can be linearized by plotting work done against time, in which case the slope of the line represents CP and the intercept represents W′, or power against 1/time, in which case the slope of the line represents W′ and the intercept represents CP.

The appropriate approach for determination of the maximal metabolic steady state (i.e., the threshold speed or power output separating heavy‐ from severe‐intensity exercise) is controversial. The ‘gold standard’ is often considered to be the so‐called maximal *lactate* steady state (MLSS; Beneke and von Duvillard 1996; Billat et al. 2003; Faude et al. 2009). The MLSS is conventionally derived from a series (typically 4–5) of 30 min continuous exercise bouts, completed on separate days, at different but constant running speeds or power outputs; blood [lactate] is measured at rest and after every 5 min of exercise and the MLSS is defined as the highest speed or power output that does not result in a rise of blood [lactate] of greater than 1 mmol/L between 10 and 30 min (Beneke 1995; Jones and Doust 1998; see Figure 2). Alternatively, the maximal metabolic steady state might be determined using the critical power (CP; or critical speed for running)1We note here that the term ‘intensity’ (e.g., ‘critical intensity’) is inappropriate in this context. This is because at a given constant speed or power output, the exercise intensity (i.e., the fraction of the $\dot{V}$O_2max_ required or the muscle metabolic perturbation evoked) can change; this is especially true for exercise in proximity to the critical speed/power where nonsteady‐state behavior is expected. It is also known that power output (the rate of energy transfer from the skeletal muscle to perform external work) and exercise intensity (the magnitude of the metabolic fluctuation(s) evoked by the task) can be completely dissociated depending on the work:recovery duration during intermittent, compared to continuous, exercise (Davies et al. 2017). It is therefore preferable to use the term ‘critical’ alongside the associated SI unit that is appropriate to the exercise modality (power, speed or velocity, torque, etc.)., which is derived from the hyperbolic relationship between speed or power output and the duration for which that speed or power output can be sustained (Hill 1925; Monod and Scherrer 1965; Hill and Smith 1999; Hill et al. 2002; Jones et al. 2010; see Figure 3).

The purpose of this article is to critique the MLSS and CP concepts and to evaluate their validity in assessing the maximal metabolic steady state. While the MLSS and CP have some conceptual similarities, tend to approximate one another and are often proposed to represent the same phenomenon, methodological differences in their assessment typically produce divergence (i.e., MLSS < CP) in practice. It is important to emphasize, therefore, that appreciating the possible advantages and disadvantages of, and the potential differences and similarities between, MLSS and CP is not merely a question of semantics. Instead, this issue is rather fundamental because it has the potential to influence performance prognosis and exercise/training prescription. It is therefore relevant not only to researchers in sport and exercise science but also to athletes, coaches, and exercise professionals. We contend that: (1) MLSS and CP should no longer be considered synonymous; and (2) CP has the more robust theoretical underpinnings and evidence base and should henceforth be considered the ‘gold standard’ for defining the maximal metabolic steady state, i.e. the boundary between the heavy‐ and severe‐intensity exercise domains.

## Considerations Regarding the Definition and Determination of MLSS

The origin of the MLSS concept is somewhat obscure but it may perhaps be attributed to the work of German physiologists, Mader and Heck, in the 1980s (Heck et al. 1985; Mader and Heck 1986). Initially, MLSS was considered to occur at a fixed blood [lactate] of 2.2 mmol/L (LaFontaine et al. 1981; Priest and Hagan 1987) or, more often, 4 mmol/L (Sjödin et al. 1982; Stegmann and Kindermann 1982; Heck et al. 1985; Mader and Heck 1986). However, discoveries that the absolute blood [lactate] at MLSS varied considerably both between individuals (Beneke and von Duvillard 1996) and between exercise modalities (Beneke et al. 2001) later led to MLSS being reconsidered to represent the speed or power output at MLSS, irrespective of the absolute blood [lactate]. Yamamoto et al. (1991) defined MLSS as the highest power output at which blood [lactate] did not increase from 15 to 30 min of continuous exercise and noted that “*In spite of the arbitrariness of the definition, the MLSS could be useful for prescribing prolonged exercise because one can exercise without continuous accumulation of blood [lactate] for at least 30 min*.” Later studies introduced a modified definition of MLSS, which remains widely used: the highest power output at which the increase in blood [lactate] is less than 1 mmol/L between 10 and 30 min of exercise (Snyder et al. 1994; Beneke and von Duvillard 1996; Jones and Doust 1998; Beneke et al. 2000). Beneke (2003) reported a protocol‐dependency of MLSS determination, with 30 min exercise bouts producing lower power output at MLSS than 20 min exercise bouts. However, the rationale for the very specific, but apparently arbitrary, definition of MLSS, including the 10–30 min timeframe and the acceptable magnitude of change in blood [lactate], is not clear.

There are several methodological concerns with the assessment of MLSS that should be highlighted. Whether a particular speed or power output is deemed to be above or below the MLSS essentially depends upon just two measurements of blood [lactate], typically made from a fingertip or earlobe blood sample, one at 10 min and the other at 30 min of exercise. If the increase in blood [lactate] is < 1 mmol/L then the speed or power output is deemed to be below MLSS, whereas if the increase is > 1 mmol/L then the speed or power output is considered to be above MLSS. It should be appreciated, however, that blood [lactate] measurement using widely used analyzers typically has an error of 0.2–0.4 mmol/L (Bonaventura et al. 2015) and that the reliability of blood [lactate] measurement, which represents a combination of both biological variation and analytical error, during submaximal exercise testing is 11–52% (Saunders et al. 2004). With such potential inaccuracy, it is obvious that the potential for false positives, i.e. that the speed or power is deemed to be above MLSS when it is not, or false negatives, i.e. that the speed or power is deemed to be below MLSS when it is not, is rather high. It should be noted also that MLSS is affected by brief interruptions in exercise that are often necessary to facilitate blood sampling (Beneke et al. 2003). Moreover, 30 min of heavy‐ to severe‐intensity exercise may result in hemoconcentration as a consequence of fluid shifts and sweating‐related dehydration which, if uncorrected, will further impact the measured [lactate], at least if measured in whole blood (Dill and Costill 1974). An added complication when exercise duration is extended for a given speed or power output within this intensity domain is the well‐known shift in substrate utilization away from carbohydrate and towards fatty acid metabolism (Hermansen et al. 1967), an adaptation which will tend to reduce muscle lactate production. Importantly, it is not certain that blood [lactate] at a given instant adequately reflects the metabolic status of the working muscle (Jorfeldt et al. 1978; Tesch et al. 1982; Bergman et al. 1999). Dynamic interaction between the rates of muscle lactate production, lactate efflux from muscle to blood, and lactate clearance/metabolism both within muscle and from the blood by other organs (Stainsby and Brooks 1990), means that a steady‐state in blood [lactate] need not imply the existence of a bioenergetic steady‐state in contracting skeletal muscle. There is also evidence that blood [lactate] dynamics can be dissociated from whole‐body oxidative metabolic rate: there are examples of elevated and rising blood [lactate] profiles in the face of a clearly steady‐state $\dot{V}$O_2_ (Scheen et al. 1981); and infusion of epinephrine has been shown to alter blood [lactate] dynamics without changing $\dot{V}$O_2_ (Gaesser et al. 1994; Womack et al. 1995). Collectively, these points suggest that blood [lactate], *per se*, is neither an appropriate nor a sufficiently sensitive metric to enable a confident assessment of whether a specific speed or power output may be sustainable in a metabolic steady‐state.

**Figure 4:**
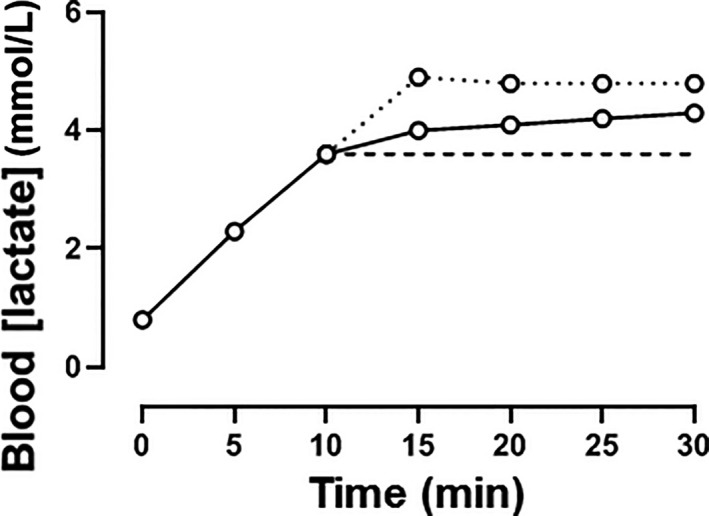
The blood [lactate] response to a constant power output test indicative of MLSS (solid black line) versus a blood [lactate] response which would be, according to the strict definition of MLSS which considers only the absolute blood [lactate] values at 10 and 30 min, defined as being above MLSS (dotted line). Note, however, that despite being supposedly *above* MLSS (dotted line), blood [lactate] stabilized between 15 and 30 min. This highlights one of the potential sources of error in defining MLSS from just two data points and applying a rather arbitrary tolerance limit (Δ1.0 mmol/L) for the increase in blood [lactate] between them.

Other aspects of the MLSS assessment protocol also merit comment. During sustained heavy‐intensity exercise, blood [lactate] tends to rise curvilinearly with time, with the rate of change of blood [lactate] tending to be greater in the first 5–10 min than in the last 5–10 min of a 30 min exercise bout (Fig. 4; Scheen et al. 1981; Jones and Doust 1998). This has the potential to lead to a scenario in which a speed or power output is deemed to be above MLSS despite blood [lactate] being stable (or even declining) over the last 10–15 min of the 30 min exercise bout, when such a profile should instead be interpreted as indicating the achievement of a delayed steady‐state. The assessment of MLSS also relies on subjects performing a series of exercise trials on different days at discrete speeds or power outputs which typically differ by 1 km/h or 10–30 W, respectively (Jones and Doust 1998; Carter et al. 1999; Smith and Jones 2001; Pringle and Jones 2002; Beneke 2003; Iannetta et al. 2018). By definition, the selection of MLSS must be at one of these discrete speeds or power outputs ‐ with the inevitable outcome that the selected MLSS must always be lower than the actual MLSS. For example, if the behavior of blood [lactate] indicates that the speed of 16 km/h is below MLSS and the speed of 17 km/h is above MLSS, then 16 km/h would be selected as the MLSS. However, had it been applied, a speed of 16.5 km/h might also have produced a blood [lactate] response consistent with exercise below MLSS such that 16.5 km/h would instead have been selected as MLSS. On average, with differences of 1 km/h or 30 W between discrete tests, the MLSS will be underestimated by 0.5 km/h for running or 15 W for cycling, respectively. The limited granularity inherent in the MLSS protocol therefore inevitably results in underestimation of the ‘actual’ MLSS. Indeed, it is crucial to appreciate that, as presently defined and measured, MLSS *must* reside *within* the heavy‐intensity domain rather than at the *boundary* of the heavy‐ and severe‐intensity domains.

The precision of the MLSS estimate is naturally enhanced by using smaller speed or power output differences from one trial to the next (e.g., 0.5 km/h for running, 15 W for cycling), but this approach is likely to increase the number of trials needed for MLSS determination. Because blood [lactate] is sensitive to changes in the hydration and nutritional status of the individual (Jacobs 1986), particularly in terms of muscle glycogen levels, subjects must refrain from normal training and consume a consistent diet over the testing period, which can extend over five days. This limits the practicality of assessing MLSS both for research purposes and in applied work with athletes. Moreover, at least in less well‐trained subjects, the lengthy protocol required for MLSS assessment might itself be sufficiently arduous that it stimulates training adaptations which result in an increased MLSS. Approaches which purport to enable accurate MLSS assessment from fewer trials have been proposed (e.g., Billat et al. 1994; Kilding and Jones 2005) but these do not obviate the other criticisms of MLSS assessment outlined above.

## Consideration of Critical Power as the Appropriate ‘Gold Standard’ for Assessing Maximal Metabolic Steady State

**Figure 5:**
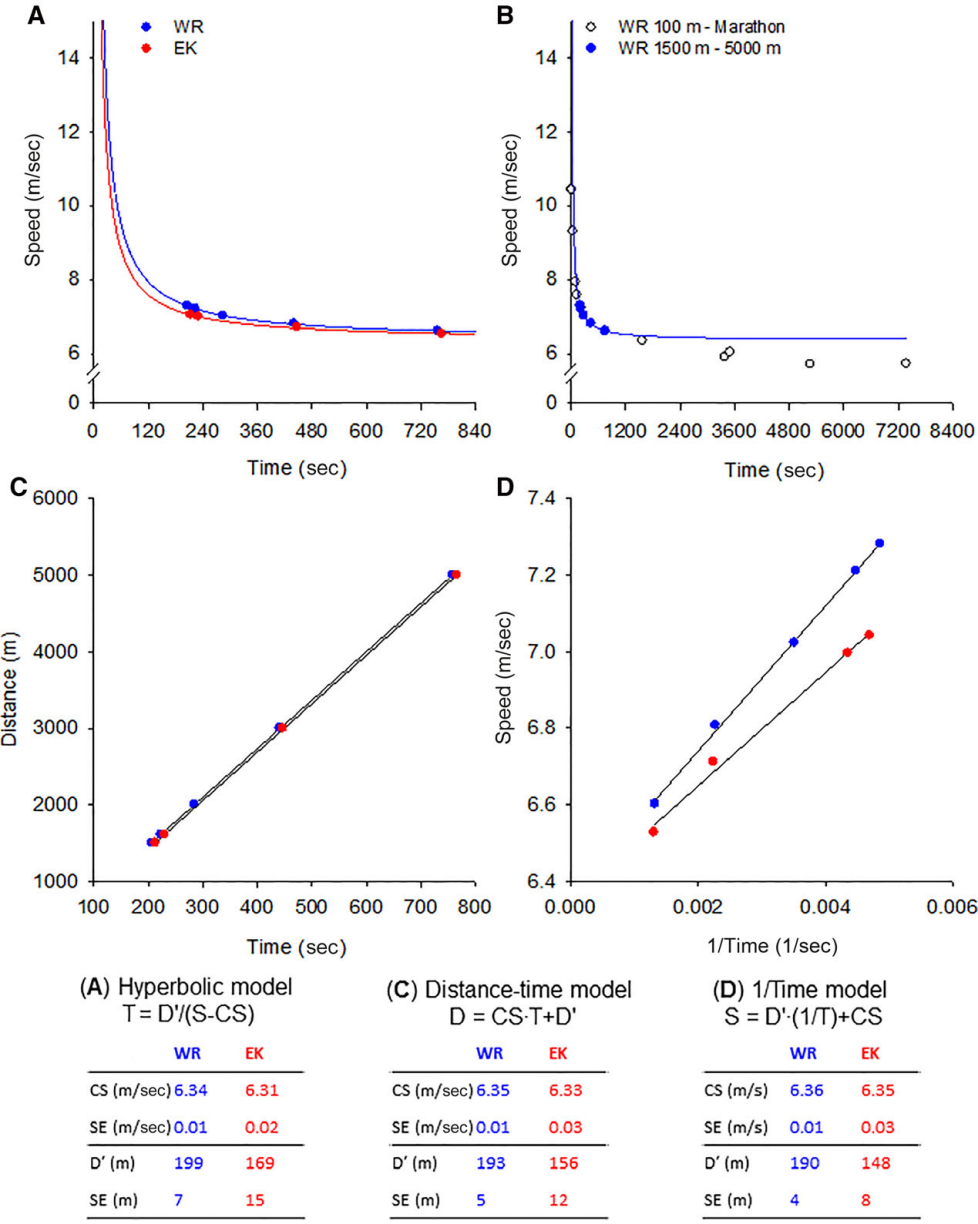
Panel A shows the hyperbolic running speed–time relationship plotted for the current (as of March 2019) world records from 1500 m to 5000 m (in blue, records held by different athletes) and the personal best times over the same distances run by an individual elite distance runner (Eliud Kipchoge, EK, in red). Panel B shows that the hyperbolic curve constructed for the world records from 1500 m to 5000 m (in blue, same data as in Panel A) does not provide a good fit to world record performances over shorter (100 m to 800 m) or longer (10,000 m to the marathon) distances. Thus, the hyperbolic relationship is valid for events which take between ~2 min and perhaps 15–20 min to complete. The linear transformation of the hyperbolic relationship is shown in Panel C (distance–time plot where the slope of the linear regression line gives critical speed, CS, and the intercept gives the curvature constant, D′) and Panel D (speed‐1/time plot where the slope gives D′ and the intercept gives CS). The CS and D′ estimates from the three equations, with the associated standard errors of the estimate, are shown at the foot of the figure.

CP has strong historical, theoretical, physiological, and mathematical foundations (Hill 1925; Wilkie 1960; Monod and Scherrer 1965; Moritani et al. 1981; Poole et al. 1988; Hill et al. 2002; Morton 2006; Jones et al. 2008; Vanhatalo et al. 2016; Mitchell et al. 2018). Indeed, the hyperbolicity of the relationship between speed or power output and the duration for which that speed or power output can be sustained was first recognized by AV Hill in 1925, following a review of world record performances in various sports (Hill 1925; see Fig. 5 for present‐day data). This hyperbolic function, with its inherent asymptote and curvature constant, is now recognized as a fundamental bioenergetic property of living systems, having been described in multiple other species (Full and Herreid 1983; Full 1986; Lauderdale and Hinchcliff 1999; Billat et al. 2005; Copp et al. 2010) and in both isolated muscle and whole body exercise modalities (Monod and Scherrer 1965; Hughson et al.^1984^; Poole et al. 1988; Burnley 2009).

**Figure 6:**
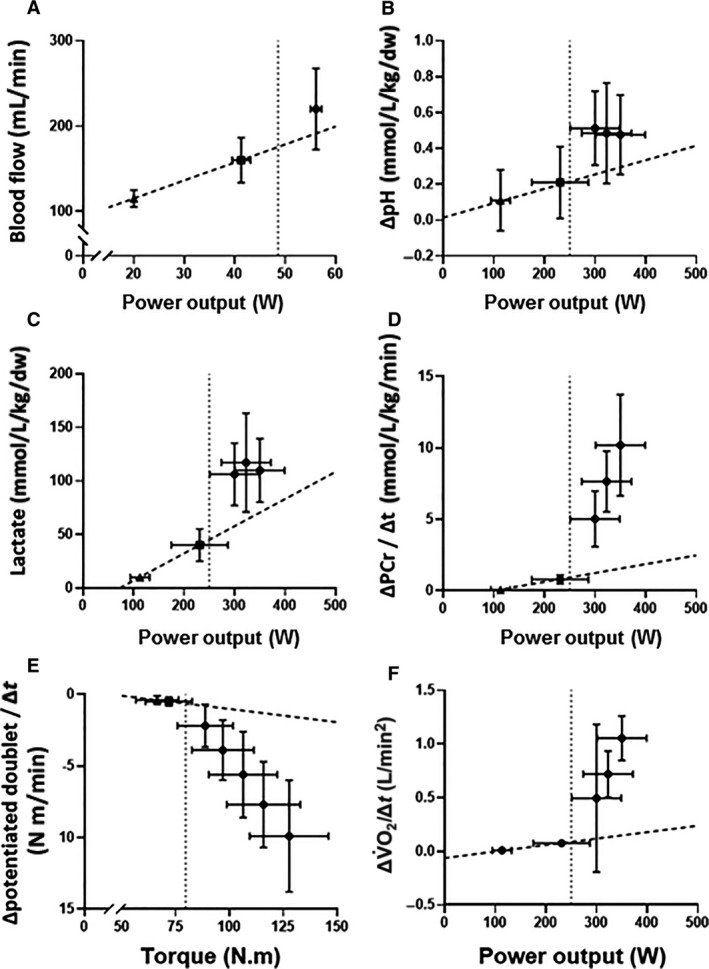
Mean ± SD muscle blood flow (panel A; Copp et al. 2010), muscle metabolic perturbation (pH, panel B; lactate, panel C; Black et al. 2017), and the rates of change in muscle [PCr] (panel D; Black et al. 2017), neuromuscular excitability (panel E; Burnley et al. 2012), and pulmonary $\dot{V}$O_2_ (panel F; Black et al. 2017) following moderate‐intensity (triangles), heavy‐intensity (squares), and severe‐intensity (circles) exercise. The dotted vertical line indicates CP, and a line of best fit has been drawn for all trials performed below CP (i.e., moderate‐ and heavy‐intensity exercise; dashed line). Note the disproportionate changes in all variables during severe‐intensity exercise (i.e., above CP) relative to exercise performed below CP. These data delineate CP as a bioenergetic threshold above which fatigue development is expedited and muscle and systemic homeostasis is precluded.

The CP is unique with regard to physiological ‘thresholds’ in that, although representing a critical metabolic rate (Barker et al. 2006; Vanhatalo et al. 2016), its definition is based purely on the measurement of mechanical work done and exercise tolerance. It is of significant interest, however, that CP separates two domains of exercise that are characterized by distinct physiological behavior. Perhaps most importantly, CP represents a boundary above which exercise results in the attainment of $\dot{V}$O_2_max, provided that exercise can be sustained for sufficiently long (i.e. ≥ approximately 2 min) for it to be reached (Poole et al. 1988; Hill and Ferguson 1999; Hill and Smith 1999; Hill et al. 2002; Vanhatalo et al. 2016). The size of the difference between the power output being sustained and CP will dictate the rate at which the finite work capacity available above CP (W′) will be utilized but, for any bout of exercise in the severe‐intensity domain, the limit of tolerance will coincide with the exhaustion of W′ and the simultaneous attainment of $\dot{V}$O_2_max (Murgatroyd et al. 2011; Vanhatalo et al. 2011b). This means that time to the limit of tolerance for any power output in the severe‐intensity domain can be accurately calculated with knowledge just of the power output to be sustained and the individual's CP and W′ (Vanhatalo et al. 2011a; Jones and Vanhatalo 2017). Moreover, unlike MLSS, in which a change in blood [lactate] is the sole index by which nonsteady‐state physiological behavior is classified, CP has been shown to separate discrete exercise intensity domains which have distinct muscle metabolic (Jones et al. 2008; Vanhatalo et al. 2016; Black et al. 2017), neuromuscular (Burnley et al. 2012; Black et al. 2017), respiratory gas exchange and ventilation (Poole et al. 1988; Murgatroyd et al. 2014), cardiovascular (Copp et al. 2010) *and* blood acid‐base (including [lactate]; Poole et al. 1988; Pringle and Jones 2002; Vanhatalo et al. 2016) profiles. These comprehensive differences in physiological behavior above and below CP are summarized in Figure 6.

Several studies have compared the independently determined MLSS and CP and reported that CP occurs at a higher power output than MLSS (Jenkins and Quigley 1990; Smith and Jones 2001; Pringle and Jones 2002; Dekerle et al. 2003, 2005; Mattioni Maturana et al. 2016; cf. Keir et al. 2015). On average, CP has been reported to be ~7% higher than MLSS (e.g., 4%, Smith and Jones 2001; 9%, Pringle and Jones 2002; 16%, Dekerle et al. 2003; 5%, Dekerle et al. 2005; 9%, Mattioni Maturana et al. 2016; cf. 1%, Keir et al. 2015). Given that MLSS will naturally underestimate the boundary between heavy‐ and severe‐intensity exercise, and taking into account the magnitude of error associated with the determination of both MLSS and CP, such a difference should not be considered surprising. However, these studies have interpreted the difference between MLSS and CP as evidence that CP does not represent the highest sustainable oxidative metabolic rate, with the inherent assumption that MLSS is the gold standard. As discussed earlier, an alternative interpretation is that the lack of agreement between MLSS and CP indicates that MLSS underestimates the highest steady‐state oxidative metabolic rate. Consistent with this, it was reported that $\dot{V}$O_2_ during continuous exercise performed at 10 W above MLSS resulted in the achievement of a steady‐state $\dot{V}$O_2_ equivalent to ~90% $\dot{V}$O_2peak_ (Mattioni Maturana et al. 2016; Iannetta et al. 2018), behavior that is clearly indicative of heavy‐intensity exercise.

An argument frequently used against CP as representing the boundary between heavy‐ and severe‐intensity exercise is that exercise tolerance falls short of a ‘fatigueless task’ when subjects are asked to exercise continuously *at* the predetermined CP (Poole et al. 1988; Jenkins and Quigley 1990; McLellan and Cheung 1992; Bull et al. 2000; Brickley et al. 2002; McClave et al. 2011; Bergstrom et al. 2013). This argument is based on a misinterpretation of the original definition of CP (Monod and Scherrer 1965) and is flawed, for two reasons. The first reason is fundamental, in that the 2‐parameter critical power model is not applicable for the prediction of exercise tolerance precisely at CP (or below it). Indeed, the tolerable limit (T_lim_) of exercise at CP would necessitate solving the following equation:

$$T_{\lim}=W^{\prime }/\left(P-\text{CP}\right),\text{where}\;P=\text{CP},=>T_{\lim}=W^{\prime }/0$$

Because Wʹ/0 is mathematically false, it is illogical to judge the validity of the CP model on the basis of an assumption of a ‘fatigueless task’ at CP. The second reason is methodological, in that performing an exercise test precisely at CP does not account for the error associated with the estimation of CP. While an advantage of the determination of the power‐duration curve is that it enables estimation of CP to a single watt, it is unreasonable to consider that this value is absolute. The approaches used to mathematically model CP will naturally be associated with some error (which is quantifiable, e.g., as standard error or 95% confidence intervals) and T_lim_ and CP will vary a little in any individual from day to day (i.e., there is some inherent biological variability; Poole et al. 1988). There is therefore a ‘bandwidth’ or ‘gray area’ surrounding the modeled CP estimate, the size of which can be minimized to approximately ± 3–5% with careful attention to protocol (see below). For example, for a CP estimate of 300 W, and a standard error of 2%, the ‘real’ CP will lie between 294 and 306 W. This means, however, that if this particular subject is exercised at exactly 300 W, there is a 50% chance that (s)he would be <CP and in the heavy‐intensity domain and a 50% chance that (s)he would be >CP and in the severe‐intensity domain. This would have important implications for physiological responses, the nature and dynamics of fatigue development, and exercise tolerance (Black et al. 2017). For this reason, it is not surprising that the time to the limit of tolerance when subjects are asked to exercise at CP is highly variable (e.g., range of approximately 15 to 40 min or occasionally up to ~60 min; McLellan and Cheung 1992; Bull et al. 2000; Brickley et al. 2002; McClave et al. 2011; Bergstrom et al. 2013), with the group mean physiological responses being characteristic of either heavy‐intensity (Poole et al. 1988, 1990; Wakayoshi et al. 1993) or severe‐intensity (Jenkins and Quigley 1990; McLellan and Cheung 1992; Brickley et al. 2002) exercise. Evidently, the practice of requiring subjects to exercise *at* CP is not an appropriate test of the validity of the concept. Indeed, the very question of ‘how long’ CP can be sustained is ill‐conceived. The crux of the matter is that CP separates an exercise domain within which physiological homeostasis can be established (heavy‐intensity domain) from one in which it cannot and in which exercise tolerance is highly predictable (severe‐intensity domain). It should also be noted that the duration of exercise *at* MLSS has never been ascertained, but this too will ultimately be unsustainable (Black et al. 2017).

Appreciation of the relationship and differences between MLSS and CP has been obfuscated by the persistent but perplexing notion that the maximal metabolic steady state should correspond to an exercise duration of approximately 1 h. This is evident in the assumption that MLSS corresponds to a so‐called ‘functional threshold’ power that can be sustained for 60 minutes (Gavin et al. 2012; Morgan et al. 2018). This is a convenient but entirely arbitrary definition that is devoid of physiological meaning. There is nothing any more ‘special’ about 60 min of exercise compared to, for example, 65 min, 44 min, or 23 min. Indeed, maximal exercise of 60 min duration is positioned squarely within the heavy‐intensity domain (Black et al. 2017) such that the physiological responses to maximal exercise of 50–55 min or 65–70 min duration, in terms of end‐exercise values and response dynamics, would likely be very similar. A more justifiable scientific approach is to define the maximal metabolic steady state as the speed or power output which separates distinct physiological response behaviors, irrespective of the corresponding exercise duration. Such an approach, which is enshrined in the CP concept, would be expected to better predict performance capability and be of greater utility in exercise/training prescription (Jones et al. 2010; Vanhatalo et al. 2011a).

It is striking that, when the standard error surrounding the estimation of CP is known and appropriately accounted for, the physiological responses to exercise performed slightly below and slightly above CP are profoundly different (Burnley et al. 2006; Jones et al. 2008; Murgatroyd et al. 2014; Vanhatalo et al. 2016). When CP is measured carefully, the standard error associated with the parameter estimate can be rather small (e.g., 4 W in Vanhatalo et al. 2007) which provides confidence that the authentic transition between an exercise domain wherein homeostasis can be (eventually) achieved from one wherein it cannot can be accurately assessed. As indicated earlier, this is true not only for pulmonary gas exchange, where a cardinal feature of severe‐intensity exercise is the development of a $\dot{V}$O_2_ ‘slow component’ that will result in the attainment of $\dot{V}$O_2max_ at or close to the point of exercise intolerance (Poole et al. 1988; Hill et al. 2002; Vanhatalo et al. 2007; Jones et al. 2010), but also for the distribution of cardiac output (Copp et al. 2010), blood acid‐base balance (Poole et al. 1998; Vanhatalo et al. 2016), and indices of muscle metabolism (e.g., muscle [PCr] and pH/lactate) whether assessed noninvasively using ^31^P‐MRS (Jones et al. 2008) or invasively via biopsy (Vanhatalo et al. 2016; Black et al. 2017). The CP therefore passes (literally) the ‘acid test’ of validity in separating the heavy‐ from the severe‐intensity exercise domains. It is important to emphasize that it is not just the absolute values of key physiological variables (e.g., $\dot{V}$O_2_, blood [lactate], muscle [PCr]) at iso‐time or end‐exercise that distinguishes severe‐ from heavy‐intensity exercise, but also the stark differences in the dynamic profiles of these and other variables (i.e., delayed steady‐state vs. nonsteady state behavior).

**Figure 7:**
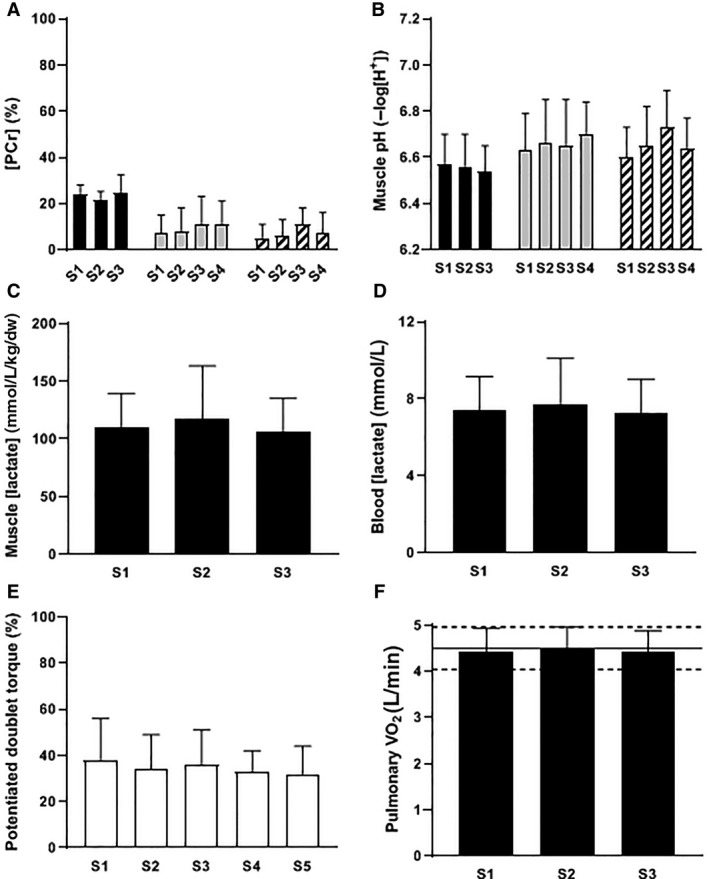
Participants performing severe‐intensity exercise attain the same “critical” muscle metabolic milieu ([PCr] panel A; pH panel B; [lactate] panel C); have similar blood [lactate] values (panel D); experience equivalent decrements in neuromuscular excitability (panel E); and achieve pulmonary $\dot{V}$O_2max_ (panel F), at the limit of tolerance irrespective of task duration. These responses are observed following cycling (black bars, Black et al. 2017) and knee‐extension exercise performed in normoxia (white bars, Burnley et al. 2012; light gray bars, Vanhatalo et al. 2010) and hyperoxia (70% O_2_, dark gray bars, Vanhatalo et al. 2010). Group mean ± SD values are displayed. *Panel F,* solid line indicates $\dot{V}$O_2max_ determined from ramp incremental test. S1 = severe‐intensity exercise bout 1, *et seq*.

Exercise at different power outputs within the severe‐intensity exercise domain results in a similar muscle metabolic status ([PCr], [Pi], pH, lactate) at the limit of tolerance (Vanhatalo et al. 2010; Black et al. 2017), consistent with the utilization of a uniform and finite W′ and the attainment of $\dot{V}$O_2_max (Murgatroyd et al. 2011; Vanhatalo et al. 2011b) (Fig. 7), whereas these variables do not show the same degree of perturbation in the heavy‐intensity domain (see Fig. 6). These results indicate that CP differentiates exercise intensity domains within which different mechanisms of fatigue development predominate (Black et al. 2017). Indeed, the available evidence suggests that exercise intolerance is associated with a relatively greater contribution from ‘peripheral’ sites during exercise >CP and a relatively greater contribution from ‘central’ factors along with muscle glycogen depletion during exercise <CP (Burnley et al. 2012; Thomas et al. 2016; Black et al. 2017). Differences in fatigue development during exercise performed below and above CP are discussed in more detail elsewhere (Poole et al. 2016; Burnley and Jones 2018). It is pertinent to reiterate here, however, that it is possible for subjects to exercise above MLSS and still produce physiological responses consistent with heavy‐intensity exercise (e.g., Mattioni Maturana et al. 2016), suggesting that MLSS does not precisely separate exercise intensity domains wherein physiological response profiles and mechanisms of fatigue development are distinct.

## Considerations for the Accurate Assessment of the Power‐Duration Relationship

While we have reviewed evidence supporting CP as *the* bona fide demarcator of the maximal metabolic steady state, it is essential that great care is taken in its estimation (Mattioni Maturana et al. 2018; Muniz‐Pumares et al. 2019). There are two methods by which CP can be assessed: the ‘conventional’ approach in which CP is modeled from a series of severe‐intensity ‘prediction trials’ performed to the limit of tolerance at different speeds or power outputs (Monod and Scherrer 1965; Poole et al. 1988); and the 3‐min all‐out test in which, as the name implies, subjects exercise maximally for 3 min with the end‐test power representing the CP and the total work done above CP representing the W′ (Burnley et al. 2006; Vanhatalo et al. 2007). If the CP is estimated using the conventional approach, important considerations include the number of trials and their duration (Hill 1993; Bishop et al. 1998; Triska et al. 2018). It is essential that subjects give their maximum effort in each trial and that cadence is consistent across all trials. Ideally the shortest trial should be 2–3 min and the longest should be more than 10 but no longer than 15 min (Hill 1993; Vanhatalo et al. 2011a). It has been recommended that there should be at least a 5 min difference between the shortest and longest trials (Bishop et al. 1998) but the goodness of hyperbolic fit is improved by making the range of times to exhaustion as broad as possible (i.e., 8–12 min) within the severe‐intensity domain. The precise duration of the prediction trials is of secondary importance to the attainment of $\dot{V}$O_2_max, but it is unusual for $\dot{V}$O_2_max to be attained if exercise duration is shorter than 1–2 min or longer than 15–20 min (Hill et al. 2002; Vanhatalo et al. 2016). $\dot{V}$O_2_ should be measured during each trial to verify attainment of $\dot{V}$O_2_max, with this typically defined as the end‐exercise $\dot{V}$O_2_ exceeding 95% of the $\dot{V}$O_2_max measured during ramp incremental exercise, to allow for biological and methodological day‐to‐day variability (Katch et al. 1982).

The goodness of fit of prediction trial data to the regression equation is dependent on the number of trials. In practice, 3‐4 (Smith and Jones 2001; Brickley et al. 2002; Pringle and Jones 2002; Dekerle et al. 2005; Black et al. 2015) or 5‐7 (Hughson et al. 1984; Gaesser and Wilson 1988; Poole et al. 1990; Bull et al. 2000; Vanhatalo et al. 2007) trials are commonly used. The goodness of fit, reported as r^2^‐values, provides only a broad indication of accuracy. The reporting of standard errors (or 95% confidence intervals) associated with each parameter is recommended, with accuracy deemed satisfactory when standard error is less than 5% of the mean for CP and less than 10% for the W′ (Hill and Smith 1994, 1999). Prediction trial data should be modeled iteratively and additional trials are performed until these SE criteria should be attained. Natural variability in human endurance performance from one test to another means that the parameter estimates derived from the three 2‐parameter models (i.e., linear work‐time model, the hyperbolic power‐time model and the linear 1/time model; Hill 1993; Morton 2006) are rarely identical. Applying all three models and finding the ‘best individual fit’ (i.e. the model which produces the least combined error for CP and W′) for each subject is a useful approach (Black et al. 2015, 2017). Variability in performance can also result in small differences in the estimated CP when a small number of prediction trials are combined, even when all trials are within the recommended range (e.g., 3, 7, and 12 min vs. 2, 5, and 10 min; Triska et al. 2018). Minor differences are to be expected given that discrete prediction trials of different durations can only provide an approximation of the underpinning fundamental power‐time continuum; such differences do not undermine the validity of CP but instead underline the importance of employing appropriate strategies to minimize measurement error.

Estimation of CP can be expedited by using the 3‐min all‐out test, which is performed by cycling against a fixed‐resistance on a Lode Excalibur Sport cycle ergometer or running on a track, and has been shown to provide valid and reliable estimates of CP (e.g., Burnley et al. 2006; Vanhatalo et al. 2007, 2008a; Pettitt et al. 2012; Broxterman et al. 2013; Simpson et al. 2015). It is important that subjects are highly motivated and fully familiarized with the protocol in its entirety, and understand that they must give a maximum effort throughout the test. Great care should be taken in the normalization of the fixed resistance for cycle ergometry. Variables measured in the 3‐min all‐out test are sensitive to manipulation of cadence (Vanhatalo et al. 2008b; Wright et al. 2019), such that selection of a ‘preferred cadence’ that is too high (≥90 rpm) tends to lead to underestimation of Wʹ and overestimation of CP. The experimenter must not provide any time‐based feedback during the test and verbal encouragement must be kept consistent to ensure that it is delivered with the same urgency and enthusiasm throughout. A substantial body of evidence indicates that, in recreationally active subjects, the peak $\dot{V}$O_2_ in the 3‐minute all‐out test typically reaches ~97–103% of ramp test determined $\dot{V}$O_2_max (Burnley et al. 2006; Vanhatalo et al. 2011b; Barker et al. 2012; Chidnok et al. 2013; Black et al. 2015; Clark et al. 2018). Hence, for the criterion test to be accepted as valid, there must be no indication of pacing in the speed or power output profile (i.e. no incremental trend in speed or power output at any point after the initial acceleration during the first 5–10 sec), and the $\dot{V}$O_2_max must be attained and then sustained for the remainder of the test (Jones et al. 2010; Vanhatalo et al. 2016). If the $\dot{V}$O_2_ attained during a 3‐minute all‐out test is < 95% $\dot{V}$O_2_max, the CP and Wʹ estimates should not be considered accurate and the test should be repeated.

## Practical Application of the Power‐Duration Relationship

Quantifying the power‐duration relationship using the testing procedures outlined above provides not just the CP (the asymptote of the relationship) but also the W′ (the curvature constant of the relationship). The CP is an index of oxidative metabolic capacity that is sensitive to endurance training (Gaesser and Wilson 1988; Poole et al. 1990; Jenkins and Quigley 1992; Vanhatalo et al. 2008a) and the fraction of inspired O_2_ (Vanhatalo et al. 2010; Dekerle et al. 2012; Simpson et al. 2015; La Monica et al. 2018). The CP is highly correlated with endurance exercise performance (Kolbe et al. 1995; Smith et al. 1999; Black et al. 2014) and it has been estimated that élite marathon runners sustain ~96% of their critical speed during competition (Jones and Vanhatalo 2017). Importantly, the W′ provides information on the finite amount of work that can be completed during exercise >CP prior to the attainment of the limit of tolerance (Fukuba et al. 2003; Chidnok et al. 2013) and is sensitive to interventions that alter the $\dot{V}$O_2_ slow component (Vanhatalo et al. 2010; Murgatroyd et al. 2011). During severe‐intensity exercise, which incorporates endurance events in the ~2–25 min range, performance is a function of the interaction of CP with W′ (Vanhatalo et al. 2011a; Jones and Vanhatalo 2017). Therefore, while CP alone provides information on the highest sustainable oxidative metabolic rate during heavy‐intensity exercise, knowing both CP and W′ enables highly accurate prediction of performance during severe‐intensity exercise (Vanhatalo et al. 2011a; Morgan et al. 2018) and is valuable in constructing individually optimized interval training programmes (Skiba et al. 2014; Jones and Vanhatalo 2017). It should be appreciated, however, that both CP and W′ are dynamic quantities that can decline with time during fatiguing exercise (Clark et al. 2018, 2019).

## Conclusions

The maximal metabolic steady state concept is valuable from multiple perspectives, such as enhancing our understanding of basic skeletal muscle energetics and fatigue processes, for characterizing exercise intensity, and for exploring and ameliorating limitations to human exercise performance. Progress in these fields has been slowed, however, by disagreement over definitions and procedures, and by a fixation with the behavior of a single biomarker, blood [lactate]. In this article, we have outlined concerns with the arbitrariness of the definition of, and the procedures for evaluating, MLSS and we have provided a rationale for considering CP as the boundary which separates steady‐state (heavy‐intensity) from nonsteady‐state (severe‐intensity) exercise. We recommend that scientists and practitioners appreciate that MLSS and CP are not, and should not be expected to be, either synonymous or interchangeable. Quantitative and qualitative differences between these entities is inevitable and are caused by the conservative definition of MLSS leading to an underestimation of the heavy/severe‐intensity boundary as represented by CP. Like all other measurements in human (exercise) physiology, there is obligatory technical error and biological variability inherent in estimating CP. However, when these are minimized by sound experimental procedure, and properly quantified and accounted for, it is evident that CP separates exercise intensity domains with distinctive muscle metabolic and systemic cardiovascular and respiratory response profiles. CP is therefore the appropriate metric when the goal is to evaluate the maximal metabolic steady state.

## Conflict of Interest

None declared.

## References

[phy214098-bib-0001] Barker, T. , D. C. Poole , M. L. Noble , and T. J. Barstow . 2006 Human critical power‐oxygen uptake relationship at different pedalling frequencies. Exp. Physiol. 91:621–632. 16527863 10.1113/expphysiol.2005.032789

[phy214098-bib-0002] Barker, A. R. , B. Bond , C. Toman , C. A. Williams , and N. Armstrong . 2012 Critical power in adolescents: physiological bases and assessment using all‐out exercise. Eur. J. Appl. Physiol. 112:1359–1370. 21805336 10.1007/s00421-011-2088-8

[phy214098-bib-0003] Beneke, R. 1995 Anaerobic threshold, individual anaerobic threshold, and maximal lactate steady state in rowing. Med. Sci. Sports Exerc. 27:863–867. 7658947

[phy214098-bib-0004] Beneke, R. 2003 Methodological aspects of maximal lactate steady state‐implications for performance testing. Eur. J. Appl. Physiol. 89:95–99. 12627312 10.1007/s00421-002-0783-1

[phy214098-bib-0005] Beneke, R. , and S. P. von Duvillard . 1996 Determination of maximal lactate steady state response in selected sports events. Med. Sci. Sports Exerc. 28:241–246. 8775160 10.1097/00005768-199602000-00013

[phy214098-bib-0006] Beneke, R. , M. Hütler , and R. M. Leithäuser . 2000 Maximal lactate‐steady‐state independent of performance. Med. Sci. Sports Exerc. 32:1135–1139. 10862542 10.1097/00005768-200006000-00016

[phy214098-bib-0007] Beneke, R. , R. M. Leithäuser , and M. Hütler . 2001 Dependence of the maximal lactate steady state on the motor pattern of exercise. Br. J. Sports Med. 35:192–196. 11375880 10.1136/bjsm.35.3.192 PMC1724327

[phy214098-bib-0008] Beneke, R. , M. Hutler , S. P. Von Duvillard , M. Sellens , and R. M. Leithauser . 2003 Effect of test interruptions on blood lactate during constant workload testing. Med. Sci. Sports Exerc. 35:1626–1630. 12972887 10.1249/01.MSS.0000084520.80451.D5

[phy214098-bib-0009] Bergman, B. C. , E. E. Wolfel , G. E. Butterfield , G. D. Lopaschuk , G. A. Casazza , M. A. Horning , et al. 1999 Active muscle and whole body lactate kinetics after endurance training in men. J. Appl. Physiol. 87:1684–1896. 10562610 10.1152/jappl.1999.87.5.1684

[phy214098-bib-0010] Bergstrom, H. C. , T. J. Housh , J. M. Zuniga , D. A. Traylor , R. W. Lewis , C. L. Camic , et al. 2013 Responses during exhaustive exercise at critical power determined from the 3‐minute all‐out test. J. Sports Sci. 31:537–545. 23121405 10.1080/02640414.2012.738925

[phy214098-bib-0011] Billat, V. , F. Dalmay , M. T. Antonini , and A. P. Chassain . 1994 A method for determining the maximal steady state of blood lactate concentration from two levels of submaximal exercise. Eur. J. Appl. Physiol. Occup. Physiol. 69:196–202. 8001529 10.1007/BF01094788

[phy214098-bib-0012] Billat, V. L. , P. Sirvent , G. Py , J. P. Koralsztein , and J. Mercier . 2003 The concept of maximal lactate steady state: a bridge between biochemistry, physiology and sport science. Sports Med. 33:407–426. 12744715 10.2165/00007256-200333060-00003

[phy214098-bib-0013] Billat, V. L. , E. Mouisel , N. Roblot , and J. Melki . 2005 Inter‐ and intrastrain variation in mouse critical running speed. J. Appl. Physiol. 98:1258–1263. 15542571 10.1152/japplphysiol.00991.2004

[phy214098-bib-0014] Bishop, D. , D. G. Jenkins , and A. Howard . 1998 The critical power function is dependent on the duration of the predictive exercise tests chosen. Int. J. Sports Med. 19:125–129. 9562222 10.1055/s-2007-971894

[phy214098-bib-0015] Black, M. I. , J. Durant , A. M. Jones , and A. Vanhatalo . 2014 Critical power derived from a 3‐minute all‐out test predicts 16.1‐km road time‐trial performance. Eur. J. Sport Sci. 14:217–223. 23802599 10.1080/17461391.2013.810306

[phy214098-bib-0016] Black, M. I. , A. M. Jones , S. J. Bailey , and A. Vanhatalo . 2015 Self‐pacing increases critical power and improves performance during severe‐intensity exercise. Appl. Physiol. Nutr. Metab. 40:662–670. 26088158 10.1139/apnm-2014-0442

[phy214098-bib-0017] Black, M. I. , A. M. Jones , J. R. Blackwell , S. J. Bailey , L. J. Wylie , S. T. McDonagh , et al. 2017 Muscle metabolic and neuromuscular determinants of fatigue during cycling in different exercise intensity domains. J. Appl. Physiol. 122:446–459. 28008101 10.1152/japplphysiol.00942.2016 PMC5429469

[phy214098-bib-0018] Bonaventura, J. M. , K. Sharpe , E. Knight , K. L. Fuller , R. K. Tanner , and C. J. Gore . 2015 Reliability and accuracy of six hand‐held blood lactate analysers. J. Sports Sci. Med. 14:203–214. 25729309 PMC4306774

[phy214098-bib-0019] Brickley, G. , J. Doust , and C. A. Williams . 2002 Physiological responses during exercise to exhaustion at critical power. Eur. J. Appl. Physiol. 88:146–151. 12436283 10.1007/s00421-002-0706-1

[phy214098-bib-0020] Broxterman, R. M. , C. J. Ade , D. C. Poole , C. A. Harms , and T. J. Barstow . 2013 A single test for the determination of parameters of the speed‐time relationship for running. Respir. Physiol. Neurobiol. 185:380–385. 22981969 10.1016/j.resp.2012.08.024

[phy214098-bib-0021] Bull, A. J. , T. J. Housh , G. O. Johnson , and S. R. Perry . 2000 Effect of mathematical modeling on the estimation of critical power. Med. Sci. Sports Exerc. 32:526–530. 10694142 10.1097/00005768-200002000-00040

[phy214098-bib-0022] Burnley, M. 2009 Estimation of critical torque using intermittent isometric maximal voluntary contractions of the quadriceps in humans. J. Appl. Physiol. 106:975–983. 19150854 10.1152/japplphysiol.91474.2008

[phy214098-bib-0023] Burnley, M. , and A. M. Jones . 2018 Power‐duration relationship: Physiology, fatigue, and the limits of human performance. Eur. J. Sport Sci. 18:1–12. 27806677 10.1080/17461391.2016.1249524

[phy214098-bib-0024] Burnley, M. , J. H. Doust , and A. Vanhatalo . 2006 A 3‐minute all‐out test to determine peak oxygen uptake and the maximal steady state. Med. Sci. Sports Exerc. 38:1995–2003. 17095935 10.1249/01.mss.0000232024.06114.a6

[phy214098-bib-0025] Burnley, M. , A. Vanhatalo , and A. M. Jones . 2012 Distinct profiles of neuromuscular fatigue during muscle contractions below and above the critical torque in humans. J. Appl. Physiol. 113:215–223. 22556396 10.1152/japplphysiol.00022.2012

[phy214098-bib-0026] Carter, H. , A. M. Jones , and J. H. Doust . 1999 Effect of 6 weeks of endurance training on the lactate minimum speed. J. Sports Sci. 17:957–967. 10622356 10.1080/026404199365353

[phy214098-bib-0027] Carter, H. , J. S. Pringle , A. M. Jones , and J. H. Doust . 2002 Oxygen uptake kinetics during treadmill running across exercise intensity domains. Eur. J. Appl. Physiol. 86:347–354. 11990749 10.1007/s00421-001-0556-2

[phy214098-bib-0028] Chidnok, W. , F. J. Dimenna , S. J. Bailey , D. P. Wilkerson , A. Vanhatalo , and A. M. Jones . 2013 Effects of pacing strategy on work done above critical power during high‐intensity exercise. Med. Sci. Sports Exerc. 45:1377–1385. 23377832 10.1249/MSS.0b013e3182860325

[phy214098-bib-0029] Clark, I. E. , A. Vanhatalo , S. J. Bailey , L. J. Wylie , B. S. Kirby , B. W. Wilkins , et al. 2018 Effects of two hours of heavy‐intensity exercise on the power‐duration relationship. Med. Sci. Sports Exerc. 50:1658–1668. 29521722 10.1249/MSS.0000000000001601

[phy214098-bib-0030] Clark, I , A. Vanhatalo , C. Thompson , L. J. Wylie , S. J. Bailey , B. Kirby , et al. 2019 Changes in the power‐duration relationship following prolonged exercise: estimation using conventional and all‐out protocols and relationship to muscle glycogen. Am. J. Physiol. Regul. Integr. Comp. Physiol.. 10.1152/ajpregu.00031.2019. [Epub ahead of print]\ 30995104

[phy214098-bib-0031] Copp, S. W. , D. M. Hirai , T. I. Musch , and D. C. Poole . 2010 Critical speed in the rat: implications for hindlimb muscle blood flow distribution and fibre recruitment. J. Physiol. 588:5077–5087. 20962004 10.1113/jphysiol.2010.198382 PMC3036198

[phy214098-bib-0032] Davies, M. J. , A. P. Benson , D. T. Cannon , S. Marwood , G. J. Kemp , H. B. Rossiter , et al. 2017 Dissociating external power from intramuscular exercise intensity during intermittent bilateral knee‐extension in humans. J. Physiol. 595:6673–6686. 28776675 10.1113/JP274589 PMC5663836

[phy214098-bib-0033] Dekerle, J. , B. Baron , L. Dupont , J. Vanvelcenaher , and P. Pelayo . 2003 Maximal lactate steady state, respiratory compensation threshold and critical power. Eur. J. Appl. Physiol. 89:281–288. 12736836 10.1007/s00421-002-0786-y

[phy214098-bib-0034] Dekerle, J. , P. Pelayo , B. Clipet , S. Depretz , T. Lefevre , and M. Sidney . 2005 Critical swimming speed does not represent the speed at maximal lactate steady state. Int. J. Sports Med. 26:524–530. 16195984 10.1055/s-2004-821227

[phy214098-bib-0035] Dekerle, J. , P. Mucci , and H. Carter . 2012 Influence of moderate hypoxia on tolerance to high‐intensity exercise. Eur. J. Appl. Physiol. 112:327–335. 21556815 10.1007/s00421-011-1979-z

[phy214098-bib-0036] Dill, D. B. , and D. L. Costill . 1974 Calculation of percentage changes in volumes of blood, plasma, and red cells in dehydration. J. Appl. Physiol. 37:247–248. 4850854 10.1152/jappl.1974.37.2.247

[phy214098-bib-0037] Faude, O. , W. Kindermann , and T. Meyer . 2009 Lactate threshold concepts: how valid are they? Sports Med. 39:469–490. 19453206 10.2165/00007256-200939060-00003

[phy214098-bib-0038] Fukuba, Y. , A. Miura , M. Endo , A. Kan , K. Yanagawa , and B. J. Whipp . 2003 The curvature constant parameter of the power‐duration curve for varied‐power exercise. Med. Sci. Sports Exerc. 35:1413–1418. 12900698 10.1249/01.MSS.0000079047.84364.70

[phy214098-bib-0039] Full, R. J. 1986 Locomotion without lungs: energetics and performance of a lungless salamander. Am. J. Physiol. 251:R775–R780. 3766777 10.1152/ajpregu.1986.251.4.R775

[phy214098-bib-0040] Full, R. J. , and C. F. 2nd Herreid . 1983 Aerobic response to exercise of the fastest land crab. Am. J. Physiol. 244:R530–R536. 6837768 10.1152/ajpregu.1983.244.4.R530

[phy214098-bib-0041] Gaesser, G. A. , and L. A. Wilson . 1988 Effects of continuous and interval training on the parameters of the power‐endurance time relationship for high‐intensity exercise. Int. J. Sports Med. 9:417–421. 3253231 10.1055/s-2007-1025043

[phy214098-bib-0042] Gaesser, G. A. , S. A. Ward , V. C. Baum , and B. J. Whipp . 1994 Effects of infused epinephrine on slow phase of O2 uptake kinetics during heavy exercise in humans. J. Appl. Physiol. 77:2413–2419. 7868463 10.1152/jappl.1994.77.5.2413

[phy214098-bib-0043] Gavin, T. P. , J. B. Van Meter , P. M. Brophy , G. S. Dubis , K. N. Potts , and R. C. Hickner . 2012 Comparison of a field‐based test to estimate functional threshold power and power output at lactate threshold. J. Strength Cond. Res. 26:416–421. 22233784 10.1519/JSC.0b013e318220b4eb

[phy214098-bib-0044] Heck, H. , A. Mader , G. Hess , S. Mücke , R. Müller , and W. Hollmann . 1985 Justification of the 4‐mmol/l lactate threshold. Int. J. Sports Med. 6:117–130. 4030186 10.1055/s-2008-1025824

[phy214098-bib-0045] Hermansen, L. , E. Hultman , and B. Saltin . 1967 Muscle glycogen during prolonged severe exercise. Acta Physiol. Scand. 71:129–139. 5584522 10.1111/j.1748-1716.1967.tb03719.x

[phy214098-bib-0046] Hill, A. V. 1925 The physiological basis of athletic records. Nature 116:544–548.

[phy214098-bib-0047] Hill, D. W. 1993 The critical power concept. A review. Sports Med. 16:237–254. 8248682 10.2165/00007256-199316040-00003

[phy214098-bib-0048] Hill, D. W. , and C. S. Ferguson . 1999 A physiological description of critical velocity. Eur. J. Appl. Physiol. Occup. Physiol. 79:290–293. 10048636 10.1007/s004210050509

[phy214098-bib-0049] Hill, D. W. , and J. C. Smith . 1994 A method to ensure accuracy of estimates of anaerobic capacity derived using the critical power concept. J. Sports Med. Physical Fitness. 34:23–37. 7934008

[phy214098-bib-0050] Hill, D. W. , and J. C. Smith . 1999 Determination of critical power by pulmonary gas exchange. Can. J. Appl. Physiol. 24:74–86. 9916183 10.1139/h99-008

[phy214098-bib-0051] Hill, D. W. , D. C. Poole , and J. C. Smith . 2002 The relationship between power and the time to achieve VO(2max). Med. Sci. Sports Exerc. 34:709–714. 11932583 10.1097/00005768-200204000-00023

[phy214098-bib-0052] Holloszy, J. O. , and E. F. Coyle . 1984 Adaptations of skeletal muscle to endurance exercise and their metabolic consequences. J. Appl. Physiol. Respir. Environ. Exerc. Physiol. 56:831–838. 6373687 10.1152/jappl.1984.56.4.831

[phy214098-bib-0053] Hughson, R. L. , C. J. Orok , and L. E. Staudt . 1984 A high velocity treadmill running test to assess endurance running potential. Int. J. Sports Med. 5:23–25. 6698679 10.1055/s-2008-1025875

[phy214098-bib-0054] Iannetta, D. , E. C. Inglis , C. Fullerton , L. Passfield , and J. M. Murias . 2018 Metabolic and performance‐related consequences of exercising at and slightly above MLSS. Scand. J. Med. Sci. Sports 28:2481–2493. 30120803 10.1111/sms.13280

[phy214098-bib-0055] Jacobs, I. 1986 Blood lactate. Implications for training and sports performance. Sports Med. 3:10–25. 2868515 10.2165/00007256-198603010-00003

[phy214098-bib-0056] Jamnick, N. A. , J. Botella , D. B. Pyne , and D. J. Bishop . 2018 Manipulating graded exercise test variables affects the validity of the lactate threshold and VO2 peak. PLoS ONE 13:e0199794. 30059543 10.1371/journal.pone.0199794 PMC6066218

[phy214098-bib-0057] Jenkins, D. G. , and B. M. Quigley . 1990 Blood lactate in trained cyclists during cycle ergometry at critical power. Eur. J. Appl. Physiol. Occup. Physiol. 61:278–283. 2282914 10.1007/BF00357613

[phy214098-bib-0058] Jenkins, D. G. , and B. M. Quigley . 1992 Endurance training enhances critical power. Med. Sci. Sports Exerc. 24:1283–1289. 1435180

[phy214098-bib-0059] Jones, A. M. , and H. Carter . 2000 The effect of endurance training on parameters of aerobic fitness. Sports Med. 29:373–386. 10870864 10.2165/00007256-200029060-00001

[phy214098-bib-0060] Jones, A. M. , and J. H. Doust . 1998 The validity of the lactate minimum test for determination of the maximal lactate steady state. Med. Sci. Sports Exerc. 30:1304–1313. 9710874 10.1097/00005768-199808000-00020

[phy214098-bib-0061] Jones, A. M. , and D. C. Poole . 2008 Physiological demands of endurance exercise Pp. 43–55 in Maughan R. J., (Ed). Olympic Textbook of Science in Sport, IOC, Blackwell, Oxford, UK.

[phy214098-bib-0062] Jones, A. M. , and A. Vanhatalo . 2017 The ‘critical power’ concept: applications to sports performance with a focus on intermittent high‐intensity exercise. Sports Med. 47:65–78. 28332113 10.1007/s40279-017-0688-0 PMC5371646

[phy214098-bib-0063] Jones, A. M. , D. P. Wilkerson , F. DiMenna , J. Fulford , and D. C. Poole . 2008 Muscle metabolic responses to exercise above and below the “critical power” assessed using ^31^P‐MRS. Am. J. Physiol. Regul. Integr. Comp. Physiol. 294:R585–R593. 18056980 10.1152/ajpregu.00731.2007

[phy214098-bib-0064] Jones, A. M. , A. Vanhatalo , M. Burnley , R. H. Morton , and D. C. Poole . 2010 Critical power: implications for determination of VO_2_max and exercise tolerance. Med. Sci. Sports Exerc. 42:1876–1890. 20195180 10.1249/MSS.0b013e3181d9cf7f

[phy214098-bib-0065] Jones, A. M. , B. Grassi , P. M. Christensen , P. Krustrup , J. Bangsbo , and D. C. Poole . 2011 Slow component of VO_2_ kinetics: mechanistic bases and practical applications. Med. Sci. Sports Exerc. 43:2046–2062. 21552162 10.1249/MSS.0b013e31821fcfc1

[phy214098-bib-0066] Jones, A. M. , M. Burnley , and A. Vanhatalo . 2018 Aerobic Exercise Performance Pp. 319–352 In Norton K., Eston R., eds. Kinanthropometry and Exercise Physiology. Routledge, London 10.4324/9781315385662

[phy214098-bib-0067] Jorfeldt, L. , A. Juhlin‐Dannfelt , and J. Karlsson . 1978 Lactate release in relation to tissue lactate in human skeletal muscle during exercise. J. Appl. Physiol. Respir. Environ. Exerc. Physiol. 44:350–352. 632175 10.1152/jappl.1978.44.3.350

[phy214098-bib-0068] Katch, V. L. , S. S. Sady , and P. Freedson . 1982 Biological variability in maximum aerobic power. Med. Sci. Sports Exerc. 14:21–25. 7070252 10.1249/00005768-198201000-00004

[phy214098-bib-0069] Keir, D. A. , F. Y. Fontana , T. C. Robertson , J. M. Murias , D. H. Paterson , J. M. Kowalchuk , et al. 2015 Exercise intensity thresholds: identifying the boundaries of sustainable performance. Med. Sci. Sports Exerc. 47:1932–1340. 25606817 10.1249/MSS.0000000000000613

[phy214098-bib-0070] Kilding, A. E. , and A. M. Jones . 2005 Validity of a single‐visit protocol to estimate the maximum lactate steady state. Med. Sci. Sports Exerc. 37:1734–1740. 16260974 10.1249/01.mss.0000181691.72432.a1

[phy214098-bib-0071] Kolbe, T. , S. C. Dennis , E. Selley , T. D. Noakes , and M. I. Lambert . 1995 The relationship between critical power and running performance. J. Sports Sci. 13:265–269. 7563294 10.1080/02640419508732236

[phy214098-bib-0072] La Monica, M. B. , D. H. Fukuda , T. M. Starling‐Smith , R. Wang , J. R. Hoffman , and J. R. Stout . 2018 Effects of normobaric hypoxia on upper body critical power and anaerobic working capacity. Respir. Physiol. Neurobiol. 249:1–6. 29247712 10.1016/j.resp.2017.12.002

[phy214098-bib-0073] LaFontaine, T. P. , B. R. Londeree , and W. K. Spath . 1981 The maximal steady state versus selected running events. Med. Sci. Sports Exerc. 13:190–193. 7253872

[phy214098-bib-0074] Lauderdale, M. A. , and K. W. Hinchcliff . 1999 Hyperbolic relationship between time‐to‐fatigue and workload. Equine Vet. J. Suppl. 30:586–590. 10.1111/j.2042-3306.1999.tb05289.x 10659323

[phy214098-bib-0075] Mader, A. , and H. Heck . 1986 A theory of the metabolic origin of anaerobic threshold. Int. J. Sports Med. 7:45–65. 3744647

[phy214098-bib-0076] Mattioni Maturana, F. , D. A. Keir , K. M. McLay , and J. M. Murias . 2016 Can measures of critical power precisely estimate the maximal metabolic steady‐state? Appl. Physiol. Nutr. Metab. 41:1197–1203. 27819154 10.1139/apnm-2016-0248

[phy214098-bib-0077] Mattioni Maturana, F. , F. Y. Fontana , S. Pogliaghi , L. Passfield , and J. M. Murias . 2018 Critical power: How different protocols and models affect its determination. J. Sci. Med. Sport. 21:742–747. 29203319 10.1016/j.jsams.2017.11.015

[phy214098-bib-0078] McClave, S. A. , M. LeBlanc , and S. A. Hawkins . 2011 Sustainability of critical power determined by a 3‐minuteute all‐out test in elite cyclists. J. Strength Cond. Res. 25:3093–3098. 21993025 10.1519/JSC.0b013e318212dafc

[phy214098-bib-0079] McLellan, T. M. , and K. S. Cheung . 1992 A comparative evaluation of the individual anaerobic threshold and the critical power. Med. Sci. Sports Exerc. 24:543–550. 1569851

[phy214098-bib-0080] Mitchell, E. A. , N. R. W. Martin , S. J. Bailey , and R. A. Ferguson . 2018 Critical power is positively related to skeletal muscle capillarity and type I muscle fibers in endurance‐trained individuals. J. Appl. Physiol. 125:737–745. 29878875 10.1152/japplphysiol.01126.2017

[phy214098-bib-0081] Monod, H. , and J. Scherrer . 1965 The work capacity of a synergic muscular group. Ergonomics 8:329–338.

[phy214098-bib-0082] Morgan, P. T. , M. I. Black , S. J. Bailey , A. M. Jones , and A. Vanhatalo . 2018 Road cycle TT performance: Relationship to the power‐duration model and association with FTP. J. Sports Sci. 37:902–910. Nov 2:1‐9. 10.1080/02640414.2018.1535772. 30387374

[phy214098-bib-0083] Moritani, T. , A. Nagata , H. A. deVries , and M. Muro . 1981 Critical power as a measure of physical work capacity and anaerobic threshold. Ergonomics 24:339–350. 7262059 10.1080/00140138108924856

[phy214098-bib-0084] Morton, R. H. 2006 The critical power and related whole‐body bioenergetic models. Eur. J. Appl. Physiol. 96:339–354. 16284785 10.1007/s00421-005-0088-2

[phy214098-bib-0085] Muniz‐Pumares, D. , B. Karsten , C. Triska , and M. Glaister . 2019 Methodological approaches and related challenges associated with the determination of critical power and curvature constant. J. Strength Cond. Res. 33:584–596. 30531413 10.1519/JSC.0000000000002977

[phy214098-bib-0086] Murgatroyd, S. R. , C. Ferguson , S. A. Ward , B. J. Whipp , and H. B. Rossiter . 2011 Pulmonary O_2_ uptake kinetics as a determinant of high‐intensity exercise tolerance in humans. J. Appl. Physiol. 110:1598–1606. 21415174 10.1152/japplphysiol.01092.2010

[phy214098-bib-0087] Murgatroyd, S. R. , L. A. Wylde , D. T. Cannon , S. A. Ward , and amd H. B. Rossiter . 2014 A ‘ramp‐sprint’ protocol to characterise indices of aerobic function and exercise intensity domains in a single laboratory test. Eur. J. Appl. Physiol. 114:1863–1874. 24888425 10.1007/s00421-014-2908-8

[phy214098-bib-0088] Pettitt, R. W. , N. Jamnick , and I. E. Clark . 2012 3‐minute all‐out exercise test for running. Int. J. Sports Med. 33:426–431. 22422309 10.1055/s-0031-1299749

[phy214098-bib-0089] Poole, D. C. , and R. S. Richardson . 1997 Determinants of oxygen uptake. Implications for exercise testing. Sports Med. 24:308–320. 9368277 10.2165/00007256-199724050-00003

[phy214098-bib-0090] Poole, D. C. , S. A. Ward , G. W. Gardner , and B. J. Whipp . 1988 Metabolic and respiratory profile of the upper limit for prolonged exercise in man. Ergonomics 31:1265–1279. 3191904 10.1080/00140138808966766

[phy214098-bib-0091] Poole, D. C. , S. A. Ward , and B. J. Whipp . 1990 The effects of training on the metabolic and respiratory profile of high‐intensity cycle ergometer exercise. Eur. J. Appl. Physiol. Occup. Physiol. 59:421–429. 2303047 10.1007/BF02388623

[phy214098-bib-0092] Poole, D. C. , M. Burnley , A. Vanhatalo , H. B. Rossiter , and A. M. Jones . 2016 Critical power: an important fatigue threshold in exercise physiology. Med. Sci. Sports Exerc. 48:2320–2334. 27031742 10.1249/MSS.0000000000000939 PMC5070974

[phy214098-bib-0093] Priest, J. W. , and R. D. Hagan . 1987 The effects of maximum steady state pace training on running performance. Br. J. Sports Med. 21:18–21. 10.1136/bjsm.21.1.18 PMC1478616 3580721

[phy214098-bib-0094] Pringle, J. S. , and A. M. Jones . 2002 Maximal lactate steady state, critical power and EMG during cycling. Eur. J. Appl. Physiol. 88:214–226. 12458364 10.1007/s00421-002-0703-4

[phy214098-bib-0095] Saunders, P. U. , D. B. Pyne , R. D. Telford , and J. A. Hawley . 2004 Reliability and variability of running economy in elite distance runners. Med. Sci. Sports Exerc. 36:1972–1976. 15514515 10.1249/01.mss.0000145468.17329.9f

[phy214098-bib-0096] Scheen, A. , J. Juchmes , and A. Cession‐Fossion . 1981 Critical analysis of the “anaerobic threshold” during exercise at constant workloads. Eur. J. Appl. Physiol. Occup. Physiol. 46:367–377. 7196326 10.1007/BF00422124

[phy214098-bib-0097] Simpson, L. P. , A. M. Jones , P. F. Skiba , A. Vanhatalo , and D. Wilkerson . 2015 Influence of hypoxia on the power‐duration relationship during high‐intensity exercise. Int. J. Sports Med. 36:113–119. 25329429 10.1055/s-0034-1389943

[phy214098-bib-0098] Sjödin, B. , I. Jacobs , and J. Svedenhag . 1982 Changes in onset of blood lactate accumulation (OBLA) and muscle enzymes after training at OBLA. Eur. J. Appl. Physiol. Occup. Physiol. 49:45–57. 6213407 10.1007/BF00428962

[phy214098-bib-0099] Skiba, P. F. , D. Clarke , A. Vanhatalo , and A. M. Jones . 2014 Validation of a novel intermittent W’ model for cycling using field data. Int. J. Sports Physiol. Perform. 9:900–904. 24509723 10.1123/ijspp.2013-0471

[phy214098-bib-0100] Smith, C. G. , and A. M. Jones . 2001 The relationship between critical velocity, maximal lactate steady‐state velocity and lactate turnpoint velocity in runners. Eur. J. Appl. Physiol. 85:19–26. 11513315 10.1007/s004210100384

[phy214098-bib-0101] Smith, J. C. , B. S. Dangelmaier , and D. W. Hill . 1999 Critical power is related to cycling time trial performance. Int. J. Sports Med. 20:374–378. 10496116 10.1055/s-2007-971147

[phy214098-bib-0102] Snyder, A. C. , T. Woulfe , R. Welsh , and C. Foster . 1994 A simplified approach to estimating the maximal lactate steady state. Int. J. Sports Med. 15:27–31. 8163322 10.1055/s-2007-1021015

[phy214098-bib-0103] Stainsby, W. N. , and G. A. Brooks . 1990 Control of lactic acid metabolism in contracting muscles and during exercise. Exerc. Sport Sci. Rev. 18:29–63. 2162774

[phy214098-bib-0104] Stegmann, H. , and W. Kindermann . 1982 Comparison of prolonged exercise tests at the individual anaerobic threshold and the fixed anaerobic threshold of 4 mmol.l(‐1) lactate. Int. J. Sports Med. 3:105–110. 7107102 10.1055/s-2008-1026072

[phy214098-bib-0105] Tesch, P. A. , W. L. Daniels , and D. S. Sharp . 1982 Lactate accumulation in muscle and blood during submaximal exercise. Acta Physiol. Scand. 114:441–446. 7136774 10.1111/j.1748-1716.1982.tb07007.x

[phy214098-bib-0106] Thomas, K. , M. Elmeua , G. Howatson , and S. Goodall . 2016 Intensity‐dependent contribution of neuromuscular fatigue after constant‐load cycling. Med. Sci. Sports Exerc. 48:1751–1760. 27187101 10.1249/MSS.0000000000000950

[phy214098-bib-0107] Triska, C. , B. Karsten , C. Beedie , B. Koller‐Zeisler , A. Nimmerichter , and H. Tschan . 2018 Different durations within the method of best practice affect the parameters of the speed‐duration relationship. Eur. J. Sport Sci. 18:332–340. 29334328 10.1080/17461391.2017.1418025

[phy214098-bib-0108] Vanhatalo, A. , J. H. Doust , and M. Burnley . 2007 Determination of critical power using a 3‐minute all‐out cycling test. Med. Sci. Sports Exerc. 39:548–555. 17473782 10.1249/mss.0b013e31802dd3e6

[phy214098-bib-0109] Vanhatalo, A. , J. H. Doust , and M. Burnley . 2008a A 3‐minute all‐out cycling test is sensitive to a change in critical power. Med. Sci. Sports Exerc. 40:1693–1699. 18685519 10.1249/MSS.0b013e318177871a

[phy214098-bib-0110] Vanhatalo, A. , J. H. Doust , and M. Burnley . 2008b Robustness of a 3 min all‐out cycling test to manipulations of power profile and cadence in humans. Exp. Physiol. 93:383–390. 17951327 10.1113/expphysiol.2007.039883

[phy214098-bib-0111] Vanhatalo, A. , J. Fulford , F. J. DiMenna , and A. M. Jones . 2010 Influence of hyperoxia on muscle metabolic responses and the power‐duration relationship during severe‐intensity exercise in humans: a 31P magnetic resonance spectroscopy study. Exp. Physiol. 95:528–540. 20028850 10.1113/expphysiol.2009.050500

[phy214098-bib-0112] Vanhatalo, A. , A. M. Jones , and M. Burnley . 2011a Application of critical power in sport. Int. J. Sports Physiol. Perform. 6:128–136. 21487156 10.1123/ijspp.6.1.128

[phy214098-bib-0113] Vanhatalo, A. , D. C. Poole , F. J. DiMenna , S. J. Bailey , and A. M. Jones . 2011b Muscle fiber recruitment and the slow component of O2 uptake: constant work rate vs. all‐out sprint exercise. Am. J. Physiol. Regul. Integr. Comp. Physiol. 300:R700–R707. 21160059 10.1152/ajpregu.00761.2010

[phy214098-bib-0114] Vanhatalo, A. , M. I. Black , F. J. DiMenna , J. R. Blackwell , J. F. Schmidt , C. Thompson , et al. 2016 The mechanistic bases of the power‐time relationship: muscle metabolic responses and relationships to muscle fibre type. J. Physiol. 594:4407–4423. 26940850 10.1113/JP271879 PMC4967754

[phy214098-bib-0115] Wakayoshi, K. , T. Yoshida , M. Udo , T. Harada , T. Moritani , Y. Mutoh , et al. 1993 Does critical swimming velocity represent exercise intensity at maximal lactate steady state? Eur. J. Appl. Physiol. Occup. Physiol. 66:90–95. 8425518 10.1007/BF00863406

[phy214098-bib-0116] Whipp, B. J. , and S. A. Ward . 1992 Pulmonary gas exchange dynamics and the tolerance to muscular exercise: effects of fitness and training. Ann. Physiol. Anthropol. 11:207–214. 1642716 10.2114/ahs1983.11.207

[phy214098-bib-0117] Wilkerson, D. P. , K. Koppo , T. J. Barstow , and A. M. Jones . 2004 Effect of work rate on the functional ‘gain’ of Phase II pulmonary O_2_ uptake response to exercise. Respir. Physiol. Neurobiol. 142:211–223. 15450481 10.1016/j.resp.2004.06.001

[phy214098-bib-0118] Wilkie, D. R. 1960 Man as a source of mechanical power. Ergonomics 3:1–8.

[phy214098-bib-0119] Womack, C. J. , S. E. Davis , J. L. Blumer , E. Barrett , A. L. Weltman , and G. A. Gaesser . 1995 Slow component of O2 uptake during heavy exercise: adaptation to endurance training. J. Appl. Physiol. 79:838–845. 8567526 10.1152/jappl.1995.79.3.838

[phy214098-bib-0120] Wright, J. , S. Bruce‐Low , and S. A. Jobson . 2019 The 3‐minuteute all‐out cycling test is sensitive to changes in cadence using the Lode Excalibur Sport ergometer. J. Sports Sci. 37:156–162. 29932805 10.1080/02640414.2018.1487115

[phy214098-bib-0121] Yamamoto, Y. , M. Miyashita , R. L. Hughson , S. Tamura , M. Shinohara , and Y. Mutoh . 1991 The ventilatory threshold gives maximal lactate steady state. Eur. J. Appl. Physiol. Occup. Physiol. 63:55–59. 1915333 10.1007/BF00760802

